# Mendelian randomisation analysis strongly implicates adiposity with risk of developing colorectal cancer

**DOI:** 10.1038/bjc.2016.188

**Published:** 2016-06-23

**Authors:** David Jarvis, Jonathan S Mitchell, Philip J Law, Kimmo Palin, Sari Tuupanen, Alexandra Gylfe, Ulrika A Hänninen, Tatiana Cajuso, Tomas Tanskanen, Johanna Kondelin, Eevi Kaasinen, Antti-Pekka Sarin, Jaakko Kaprio, Johan G Eriksson, Harri Rissanen, Paul Knekt, Eero Pukkala, Pekka Jousilahti, Veikko Salomaa, Samuli Ripatti, Aarno Palotie, Heikki Järvinen, Laura Renkonen-Sinisalo, Anna Lepistö, Jan Böhm, Jukka-Pekka Meklin, Nada A Al-Tassan, Claire Palles, Lynn Martin, Ella Barclay, Susan M Farrington, Maria N Timofeeva, Brian F Meyer, Salma M Wakil, Harry Campbell, Christopher G Smith, Shelley Idziaszczyk, Timothy S Maughan, Richard Kaplan, Rachel Kerr, David Kerr, Daniel D Buchanan, Aung K Win, John L Hopper, Mark A Jenkins, Noralane M Lindor, Polly A Newcomb, Steve Gallinger, David Conti, Fred Schumacher, Graham Casey, Jussi Taipale, Lauri A Aaltonen, Jeremy P Cheadle, Malcolm G Dunlop, Ian P Tomlinson, Richard S Houlston

**Affiliations:** 1 Division of Genetics and Epidemiology, The Institute of Cancer Research, London SW7 3RP, UK; 2 Genome-Scale Biology Research Program, Research Programs Unit, University of Helsinki 00014, Helsinki, Finland; 3 Department of Medical and Clinical Genetics, Medicum, University of Helsinki, Helsinki 00014, Finland; 4 Institute for Molecular Medicine Finland, University of Helsinki, Helsinki 00014, Finland; 5 National Institute for Health and Welfare, Helsinki 00271, Finland; 6 Folkhälsan Research Centre, Helsinki 00250, Finland; 7 Unit of General Practice and Primary Health Care, University of Helsinki, Helsinki University Hospital, Helsinki 00014, Finland; 8 Finnish Cancer Registry, Institute for Statistical and Epidemiological Cancer Research, Helsinki 00130, Finland; 9 School of Health Sciences, University of Tampere, Tampere 33014, Finland; 10 Department of Medicine, Analytic and Translational Genetics Unit, Massachusetts General Hospital, Boston, MA 02114, USA; 11 Program in Medical and Population Genetics, The Broad Institute of MIT and Harvard, Cambridge, MA 02142, USA; 12 Department of Neurology, Massachusetts General Hospital, Boston, MA 02114, USA; 13 Department of Surgery, Helsinki University Central Hospital, Hospital District of Helsinki and Uusimaa, Helsinki 00029, Finland; 14 Department of Surgery, Abdominal Center, Helsinki University Hospital Helsinki 00029, Finland; 15 Department of Pathology, Central Finland Central Hospital, Jyväskylä 40620, Finland; 16 Department of Surgery, Jyväskylä Central Hospital, University of Eastern Finland, Jyväskylä 40620, Finland; 17 Department of Genetics, King Faisal Specialist Hospital and Research Center, Riyadh 12713, Saudi Arabia; 18 Wellcome Trust Centre for Human Genetics, NIHR Comprehensive Biomedical Research Centre, Oxford OX3 7BN, UK; 19 Colon Cancer Genetics Group, MRC Human Genetics Unit, The University of Edinburgh, Western General Hospital, Edinburgh EH4 2XU, UK; 20 The Roslin Institute, University of Edinburgh, Easter Bush, Roslin, Edinburgh EH25 9RG, UK; 21 Centre for Population Health Sciences, University of Edinburgh, Edinburgh EH8 9AG, UK; 22 Institute of Cancer and Genetics, School of Medicine, Cardiff University, Cardiff CF14 4XN, UK; 23 CRUK/MRC Oxford Institute for Radiation Oncology, University of Oxford, Oxford OX3 7DQ, UK; 24 MRC Clinical Trials Unit, Aviation House, London WC2B 6NH, UK; 25 Department of Oncology, Oxford Cancer Centre, University of Oxford, Churchill Hospital, Oxford OX3 7LE, UK; 26 Nuffield Department of Clinical Laboratory Sciences, University of Oxford, John Radcliffe Hospital, Oxford OX3 9DU, UK; 27 Department of Pathology, Colorectal Oncogenomics Group, Genetic Epidemiology Laboratory, The University of Melbourne, Melbourne, VIC 3010, Australia; 28 Centre for Epidemiology and Biostatistics, The University of Melbourne, Melbourne, VIC 3010, Australia; 29 Department of Health Sciences Research, Mayo Clinic, Scottsdale, AZ 85259, USA; 30 Cancer Prevention Program, Fred Hutchinson Cancer Research Center, Seattle, WA 98109, USA; 31 Lunenfeld-Tanenbaum Research Institute, Mount Sinai Hospital, Toronto, ON M5G 1X5, Canada; 32 Department of Preventive Medicine, University of Southern California, Los Angeles, CA 90033, USA; 33 Department of Biosciences and Nutrition, SciLife Center, Karolinska Institutet, Solna SE 141 83, Sweden

**Keywords:** Mendelian randomisation, adiposity, colorectal cancer

## Abstract

**Background:**

Observational studies have associated adiposity with an increased risk of colorectal cancer (CRC). However, such studies do not establish a causal relationship. To minimise bias from confounding we performed a Mendelian randomisation (MR) analysis to examine the relationship between adiposity and CRC.

**Methods:**

We used SNPs associated with adult body mass index (BMI), waist-hip ratio (WHR), childhood obesity and birth weight as instrumental variables in a MR analysis of 9254 CRC cases and 18 386 controls.

**Results:**

In the MR analysis, the odds ratios (ORs) of CRC risk per unit increase in BMI, WHR and childhood obesity were 1.23 (95% CI: 1.02–1.49, *P*=0.033), 1.59 (95% CI: 1.08–2.34, *P*=0.019) and 1.07 (95% CI: 1.03–1.13, *P*=0.018), respectively. There was no evidence for association between birth weight and CRC (OR=1.22, 95% CI: 0.89–1.67, *P*=0.22). Combining these data with a concurrent MR-based analysis for BMI and WHR with CRC risk (totalling to 18 190 cases, 27 617 controls) provided increased support, ORs for BMI and WHR were 1.26 (95% CI: 1.10–1.44, *P*=7.7 × 10^−4^) and 1.40 (95% CI: 1.14–1.72, *P*=1.2 × 10^−3^), respectively.

**Conclusions:**

These data provide further evidence for a strong causal relationship between adiposity and the risk of developing CRC highlighting the urgent need for prevention and treatment of adiposity.

Colorectal cancer (CRC) is a major public health problem in economically developed countries (Forman *et al*, 2014). Migration and epidemiological studies have established that diet and other lifestyle factors have a major role in the aetiology of CRC (Haggar and Boushey, 2009). Among the lifestyle factors that have an impact on CRC risk, high body mass index (BMI) has generally been reported to be associated with an increased risk in observational studies (Bianchini *et al*, 2002; Bardou *et al*, 2013), with some evidence for a stronger influence being shown for men (Pan *et al*, 2004; Moghaddam *et al*, 2007). There is also evidence for an inverse relationship between adiposity at a young age and cancer development (Carpenter *et al*, 2003; Yang *et al*, 2014). The association between BMI and CRC in these observational studies does not, however, necessarily establish a causal relationship. Specifically, these studies cannot entirely exclude the possibility of the observed association being the consequence of confounding factors, such as socio-economic status, alcohol and other lifestyle factors (Moghaddam *et al*, 2007), whereas some studies have failed to exclude the possibility of reverse causation (Smith and Ebrahim, 2003).

An alternative to a traditional observational epidemiology study is the Mendelian randomisation (MR) approach. The strategy of MR uses genetic markers known to be associated with a potential risk factor in the assessment of its effect on another trait or disease (Lawlor *et al*, 2008). These markers, or instrumental variables (IV), rely on a number of assumptions, namely that the IVs are solely associated with the trait or disease, and the IVs are independent of confounders. This methodology permits the nature of the relation between the risk factor and the trait or disease to be assessed without the limitations present within observational studies, such as confounding factors, and importantly establish whether an association is causal. A further attribute of MR is the avoidance of the influence of factors whose effect may be time sensitive, for example weight loss being a consequence of CRC. Thus, an IV has the potential to more accurately assess lifetime exposure when compared with measurements of potential risk factors recorded in an observational study.

In the present study we examined, using MR, the impact of four metrics of adiposity on the risk of developing CRC. The adiposity traits we considered were adult BMI, adult waist-hip ratio (WHR), childhood obesity and birth weight.

## Materials and methods

### CRC GWAS data sets

Our MR analysis was based on data from seven previously reported genome-wide association studies (GWAS) of CRC (Orlando *et al*, 2016). Briefly, these GWAS were all based on individuals with European ancestry and comprise: CCFR1 (1290 cases, 1055 controls), CCFR2 (796 cases, 2236 controls), COIN (2244 cases, 2162 controls), FINLAND (1172 cases, 8266 controls), UK1 (940 cases, 965 controls), Scotland1 (1012 cases, 1012 controls) and VQ58 (1800 cases, 2690 controls). Details of the genotyping, quality control and imputation of untyped single-nucleotide polymorphisms (SNPs) genotypes have been previously published. Summary statistics from the GWAS were used to calculate the ratio estimates for the adiposity-related SNPs.

### Instrumental variables

For each of four adiposity traits (adult BMI, adult WHR, childhood obesity and birth weight), we used data from recent GWAS of individuals of European-decent for each trait. Specifically for adult BMI, we used data from the Genetic Investigation of Anthropometric Traits (GIANT) consortium, which was based on an analysis of up to 339 224 individuals (Locke *et al*, 2015). For the WHR, data were obtained from a meta-analysis comprising 61 studies, made up of up to 190 803 individuals (Heid *et al*, 2010). For childhood obesity, data was obtained from 2480 children with extreme obesity and 7370 controls (Wheeler *et al*, 2013). Birth weight data was obtained from up to 69 308 individuals from 43 studies (Horikoshi *et al*, 2013).

We used SNPs that were declared genome-wide significant (i.e., *P*⩽5.0 × 10^−8^) in these GWAS as IVs. From the 97 adult BMI-associated SNPs (kg m^−2^) as identified by Locke *et al* (2015), we confined the analysis to 76 SNPs that were found in European populations, and were separated by at least 500 kb. In addition, we used 14 SNPs for WHR as reported by Heid *et al* (2010); nine SNPs reported by Wheeler *et al* (2013), which were associated with childhood obesity (>3 s.d. from the mean of the BMI distribution); and the seven SNPs reported by Horikoshi *et al* (2013), which were associated with birth weight (kg) (Supplementary Table 1). None of these studies reported non-additive effects of the SNPs on the adiposity trait, hence per allele effects were considered additive. For each of the four adiposity traits we extracted the effect estimates and associated *P*-values of each SNP from the seven CRC GWAS.

### Statistical analysis

We performed MR analysis to assess the association between each adiposity trait and CRC using summary statistics from the CRC GWAS, and the published effect size of the adiposity-related trait on CRC. As per Burgess *et al* (2015) the combined ratio estimate 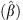 of all SNPs associated with a particular adiposity trait on CRC risk was calculated under a fixed-effects model:


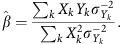


*X*_*k*_ corresponds to the association between SNP *k* with the adiposity trait and *Y*_*k*_ is the association between SNP *k* and CRC risk with standard error 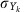. The standard error of the combined ratio estimate is approximately given by


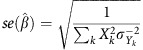


We used MR-Egger and inverse weighted variance regression to examine for violation (e.g., from pleiotopy) of the standard IV assumptions in our analysis (Bowden *et al*, 2015). All analyses were performed using R software (R Development Core Team, Vienna, Austria) and we considered a *P*-value of <0.05 as significant.

### Meta-analysis with published studies

An overlapping concurrent study has recently been published which has also reported an MR analysis to estimate the causal association between adiposity and CRC risk (Thrift *et al*, 2015). The study was based on the analysis of 10 226 CRC cases and 10 286 controls from 11 GWAS, including the CCFR1 study, reporting on the impact of adult BMI and WHR on CRC. The genetic risk score for these two adiposity traits in this study and in our analysis was based on the same set of SNPs. Hence, to enhance our power to establish a relationship between genetically defined adiposity and CRC risk, we performed a meta-analysis of our study and this published study pooling summary estimates of effect size under a fixed-effects model, avoiding duplication of the CCFR1 study.

## Results

**Figure 1:**
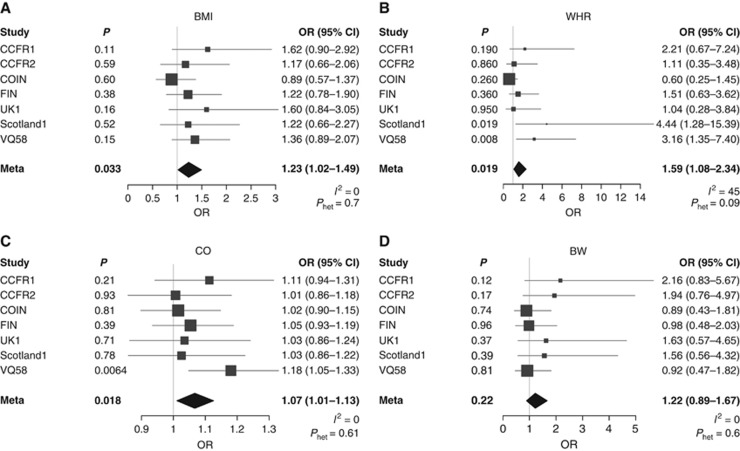
**MR results of adiposity traits on CRC risk from seven GWAS studies and the meta-analysis.** (**A**) Adult BMI; (**B**) adult WHR; (**C**) childhood obesity (CO); and (**D**) birth weight (BW). Boxes denote OR point estimates, with their areas proportional to the inverse variance weight of the estimate. Horizontal lines represent 95% CIs. The diamond represents the summary Ors, computed under a fixed-effects model, with 95% CI given by the width of the diamond. The unbroken vertical line is at the null value (OR=1.0).

In six of the seven CRC GWAS there was a positive relationship between BMI-increasing alleles and CRC risk (Figure 1). From the pooled analysis we identified an odds ratio (OR) of 1.23 in risk of CRC per kg m^−2^ increase of BMI (95% confidence interval (CI): 1.02–1.49, *P*=0.033, test for heterogeneity between studies *I*^2^=0%, *P*_het_=0.70).

Although the relationship between WHR and childhood obesity with CRC was less consistent between studies than for BMI in the pooled analysis, we also identified a correlation between both adiposity traits and CRC risk (Figure 1). For each unit increase in WHR we observed an OR of 1.59 in CRC risk (95% CI: 1.08–2.34, *P*=0.019 test for heterogeneity across studies *I*^2^=45%, *P*_het_=0.09). For childhood obesity the OR for CRC was 1.07 (95% CI: 1.01–1.13, *P*=0.018, test for heterogeneity between studies *I*^2^=0%, *P*_het_=0.61). In contrast to the relationship between BMI, WHR, and childhood obesity and CRC we observed no association with birth weight and risk (*P*=0.22, Figure 1).

Using both MR-Egger and IVW regression tests we did not detect violation of the standard IV assumptions in our MR analysis of BMI, WHR, childhood obesity or birth weight for CRC risk (Supplementary Figure 1, Supplementary Table 2).

The strongest reported SNP associations for BMI are provided by rs1558902 (*FTO*) and rs13021737 (*TMEM18*), which have a well-established impact on obesity (Frayling *et al*, 2007; Rohde *et al*, 2014). To examine if our findings of a correlation between BMI and CRC risk were primarily driven by these variants we performed a sensitivity analysis excluding these SNPs. The MR results remain statistically significant, albeit slightly less profound (OR=1.24, 95% CI: 1.01–1.51, *P*=0.035). The association between each of the adiposity-related SNP and CRC risk is shown in Supplementary Figure 1.

Given there is correlation between measures of adiposity (Serdula *et al*, 1993) we examined the specificity of each trait on CRC risk by repeating our analysis omitting SNPs if there were overlapping loci. With the exception of adult BMI and childhood obesity which share four associated loci *FTO* (rs1558902, rs1421085), *MC4R* (rs6567160, rs476828), *TMEM18* (rs13021737, rs12463617) and *NEGR1* (rs3101336) all of the adiposity-related SNPs are distinct. Omitting these overlapping SNPs from our MR analysis still provided evidence for a correlation between adult BMI and childhood obesity with CRC risk, albeit less significant than before (OR=1.23, 95% CI: 1.00–1.51, *P*=0.049 and OR=1.10, 95% CI: 1.02–1.20, *P*=0.019, respectively).

To explore if there were gender-specific associations for CRC for each of the four adiposity traits, we performed a stratified analysis of our data set. Although an increasing number of risk alleles for each trait was associated with an increased CRC in both men and women, only the relationship between adult WHR and CRC in men remained statistically significant (Supplementary Table 3; OR=2.13, 95% CI: 1.18–3.87, *P*=0.013).

In the meta-analysis combining these results with the data from Thrift *et al* (2015), adult BMI was associated with an OR of 1.26 for CRC risk (95% CI: 1.10–1.44, *P*=7.7 × 10^−4^) and adult WHR an OR of 1.40 for CRC risk (95% CI: 1.14–1.72, *P*=1.2 × 10^−3^; Table 1). Although the association between genetically influenced adult BMI was only significant in women (ORs in females and males were 1.43, 95% CI: 1.17–1.74, *P*=4.3 × 10^−4^ and 1.12, 95% CI: 0.92–1.38, *P*=0.26, respectively) and the WHR association was only significant for men (ORs in males and females were 1.63, 95% CI: 1.20–2.22, *P*=2.0 × 10^−3^ and 1.22, 95% CI: 0.91–1.63, *P*=0.18, respectively) these differences were not statistically different (*P*-values for difference were 0.09 and 0.18, respectively).

## Discussion

In this study we have shown correlations between IVs for adult BMI, WHR and childhood obesity, and CRC risk. Even adjusting for multiple testing the correlations between IVs for adult BMI and WHR remained significant. The absence of data on childhood obesity in the study reported by Thrift *et al* (2015), precluded meta-analysis of this metric of adiposity. We did not identify a relationship between the IVs for birth weight and CRC. Although this is less likely to be a determinant of CRC risk *per se*, we acknowledge that our power to demonstrate a relationship was limited. Indeed, even the IVs for adult BMI we used explain ∼3% of the total phenotypic variation in BMI (Locke *et al*, 2015).

A possible explanation for a failure to demonstrate a significant correlation between genetic BMI and CRC risk could be that the distribution of body fat is a more important predictor of CRC risk in men rather than total body adiposity. Support for this postulate is that in men WHR has been reported to be superior in predicting CRC than BMI in observational studies. In our MR-based analysis we indeed found that WHR was associated with CRC in men but not in women. Given that the genetic risk score for WHR is derived from SNPs associated with WHR, which are adjusted for BMI, suggests that fat distribution may be important for CRC risk for men, whereas overall obesity is more important for CRC risk for women.

There is evidence that adiposity may have a more significant effect on the development of colon rather than rectal cancer. Given that the landscape of colonic and rectal cancers show differences relating IVs to molecular features is likely to be informative in terms of understanding disease aetiology (Yamauchi *et al*, 2012). Unfortunately the data sets on which our study has been based did not enable such analysis to be performed.

An important strength of our analysis is that by implementing an MR-based analysis we have avoided the potential biases in observational studies of factors such as recall bias and confounding. The combined ratio estimates of the impact of adiposity on CRC risk hold provided the marker is independent of factors that may confound the relationship between adiposity and CRC. The findings from our analysis are however reliant on a number of key assumptions. First, that the IVs are solely associated with CRC through its association with adiposity rather than pleiotropism, which would be seen by a departure from linearity of the relationship between SNPs and their effect size for adiposity and CRC. We did not observe such a relationship between CRC and adiposity risk SNPs. Second, the IV is independent of factors that confound observational associations. To date there is currently no evidence that the IVs we used are associated with factors that might confound adiposity CRC associations in conventional analyses. The generation of substantive IVs for highly complex traits is a major limitation of the MR-based strategy to investigate the aetiological basis of diseases like cancer.

Accepting these caveats our study findings can be viewed as quantifying the causal effect of adiposity on CRC risk. Moreover, they generally support previously published observational studies and provide further evidence for adiposity being a major risk factor for the development of CRC.

In the pooled analysis, genetically influenced BMI showed only a statistically significant association with CRC in women. This finding is discordant with observational studies which have generally found a stronger correlation in men. It is possible that some estimates from observational studies may have been biased toward the null if heavier women under-report their weight. Indeed it is noteworthy that this specific finding from the meta-analysis is primarily driven by data from Thrift *et al* (2015), hence independent replication is required.

The biological mechanism by which adiposity increases CRC risk remains to be established and several mechanisms have been variously suggested as explaining the correlation. These include increased insulin and insulin-like growth factor signalling, chronic inflammation and signalling via adipokines, such as leptin. Furthermore, it is plausible that increased adiposity may alter the intestinal microbiome, contributing to gastrointestinal carcinogenesis (Harriss *et al*, 2009; Kant and Hull, 2011; Aleman *et al*, 2014). Irrespective of the exact functional basis of the association between adiposity and CRC risk, demonstrating that it is causal makes obesity an important target for primary prevention of CRC in the population.

## Figures and Tables

**Table 1:** MR results showing the relationship between CRC risk, and adult BMI and adult WHR in the meta-analysis with published data

	**BMI**		**WHR**	
**Sex**	**OR (95% CI)**	***P*** **value**	**OR (95% CI)**	***P*** **value**
All	1.26 (1.10–1.44)	7.73 × 10^−4^	1.40 (1.14–1.72)	1.22 × 10^−3^
Male	1.12 (0.92–1.38)	0.262	1.63 (1.20–2.22)	1.98 × 10^−3^
Female	1.43 (1.17–1.74)	4.25 × 10^−4^	1.22 (0.91–1.63)	0.178

Abbreviations: MR=Mendelian randomisation; CRC=colorectal cancer; BMI=body mass index; WHR=waist-hip ratio; OR=odds ratio; CI=confidence interval.
