# Supplementary material for: Mendelian randomisation analysis strongly implicates adiposity with risk of developing colorectal cancer
Source: Br J Cancer. 2016 Jun 23;115(2):266–72. doi: 10.1038/bjc.2016.188 (PMC4947703; doi:10.1038/bjc.2016.188)
Supplement: Supplementary Information [file bjc2016188x1.docx]

**Supplementary Material**

**Mendelian randomization analysis strongly implicates adiposity with risk of developing colorectal cancer**

David Jarvis, Jonathan Mitchell, Philip Law, Kimmo Palin, Sari Tuupanen, Alexandra Gylfe, Ulrika A. Hänninen, Tatiana Cajuso, Tomas Tanskanen, Johanna Kondelin, Eevi Kaasinen, Antti-Pekka Sarin, Jaakko Kaprio, Johan G. Eriksson, Harri Rissanen, Paul Knekt, Eero Pukkala, Pekka Jousilahti, Veikko Salomaa, Samuli Ripatti, Aarno Palotie, Heikki Järvinen, Laura Renkonen-Sinisalo, Anna Lepistö, Jan Böhm, Jukka-Pekka Meklin, Nada A. Al-Tassan, Claire Palles, Lynn Martin, Ella Barclay, Susan M. Farrington, Maria N. Timofeeva, Brian F. Meyer, Salma M. Wakil, Harry Campbell, Christopher G. Smith, Shelley Idziaszczyk, Timothy S. Maughan, Richard Kaplan, Rachel Kerr, David Kerr, Daniel D. Buchanan, Aung K. Win, John L. Hopper, Mark A. Jenkins, Noralane M. Lindor, Polly A. Newcomb, Steve Gallinger, David Conti, Fred Schumacher, Graham Casey, Jussi Taipale, Lauri A. Aaltonen, Jeremy P. Cheadle, Malcolm G. Dunlop, Ian P. Tomlinson, Richard S. Houlston

**Supplementary Figure 1: Adiposity SNP-specific effects for risk of colorectal cancer.** For each figure, the effect size of the respective adiposity effect is plotted against the effect of colorectal cancer. Each point represents a SNP that was found to be associated with the adiposity effect. The red line represents a linear regression line through the points. A greater slope is indicative of a pleotropic effect.

(A) Adult body-mass index

SNPs from left to right, top to bottom:

rs6477694, rs10733682, rs11688816, rs1808579, rs3736485, rs11583200, rs29941, rs4740619, rs1528435, rs12940622, rs2033529, rs3849570, rs9925964, rs1928295, rs9400239, rs2033732, rs17724992, rs1000940, rs6804842, rs2820292, rs2365389, rs2176598, rs1167827, rs12885454, rs4256980, rs12401738, rs11126666, rs2650492, rs7243357, rs11165643, rs17405819, rs12286929, rs7599312, rs205262, rs7903146, rs657452, rs1016287, rs10132280, rs758747, rs7141420, rs12566985, rs17094222, rs2121279, rs10968576, rs3817334, rs2075650, rs2112347, rs13191362, rs3810291, rs13078960, rs16951275, rs17001654, rs11191560, rs10182181, rs3888190, rs11057405, rs7138803, rs2245368, rs12429545, rs3101336, rs2287019, rs11727676, rs10938397, rs12446632, rs7899106, rs11030104, rs2207139, rs1516725, rs16851483, rs13107325, rs543874, rs11847697, rs6567160, rs13021737, rs17024393

(B) Adult waist-hip ratio

(C) Childhood obesity

(D) Birth weight

**Supplementary Table 1: Effect size and standard error for each adiposity trait and CRC risk for SNPs used as instrumental variables.**

(A) Adult body-mass index (BMI)

|  |  |  |  |  |  |  |  |  |  |
| --- | --- | --- | --- | --- | --- | --- | --- | --- | --- |
| RSID | BMI ß | BMI SE | CRC ß | CRC SE | RSID | BMI ß | BMI SE | CRC ß | CRC SE |
|  |  |  |  |  |  |  |  |  |  |
| rs6567160 | 0.056 | 0.004 | 0.025 | 0.024 | rs7903146 | 0.023 | 0.003 | -0.0092 | 0.022 |
| rs13021737 | 0.060 | 0.004 | 0.010 | 0.027 | rs10132280 | 0.023 | 0.003 | 0.017 | 0.024 |
| rs10938397 | 0.040 | 0.003 | -0.0093 | 0.020 | rs17094222 | 0.025 | 0.004 | 0.056 | 0.022 |
| rs543874 | 0.048 | 0.004 | -0.013 | 0.025 | rs7599312 | 0.022 | 0.003 | -0.0086 | 0.020 |
| rs2207139 | 0.045 | 0.004 | 0.0026 | 0.026 | rs2365389 | 0.020 | 0.003 | -0.032 | 0.020 |
| rs11030104 | 0.041 | 0.004 | -0.079 | 0.024 | rs2820292 | 0.020 | 0.003 | 0.079 | 0.021 |
| rs3101336 | 0.033 | 0.003 | -0.0037 | 0.020 | rs12885454 | 0.021 | 0.003 | -0.016 | 0.041 |
| rs7138803 | 0.032 | 0.003 | 0.021 | 0.021 | rs16851483 | 0.048 | 0.008 | 0.074 | 0.020 |
| rs10182181 | 0.031 | 0.003 | -0.0080 | 0.020 | rs1167827 | 0.020 | 0.003 | -0.00084 | 0.023 |
| rs3888190 | 0.031 | 0.003 | -0.014 | 0.020 | rs758747 | 0.023 | 0.004 | -0.027 | 0.020 |
| rs1516725 | 0.045 | 0.005 | -0.043 | 0.029 | rs1928295 | 0.019 | 0.003 | -0.018 | 0.021 |
| rs12446632 | 0.040 | 0.005 | 0.020 | 0.028 | rs9925964 | 0.019 | 0.003 | -0.026 | 0.022 |
| rs2287019 | 0.036 | 0.004 | 0.036 | 0.026 | rs11126666 | 0.021 | 0.003 | -0.025 | 0.023 |
| rs16951275 | 0.031 | 0.004 | -0.084 | 0.024 | rs2650492 | 0.021 | 0.004 | -0.027 | 0.020 |
| rs3817334 | 0.026 | 0.003 | -0.0035 | 0.020 | rs6804842 | 0.019 | 0.003 | -0.035 | 0.020 |
| rs2112347 | 0.026 | 0.003 | 0.037 | 0.021 | rs4740619 | 0.018 | 0.003 | -0.0069 | 0.032 |
| rs12566985 | 0.024 | 0.003 | -0.0061 | 0.020 | rs13191362 | 0.028 | 0.005 | -0.033 | 0.020 |
| rs3810291 | 0.028 | 0.004 | -0.054 | 0.022 | rs3736485 | 0.018 | 0.003 | -0.057 | 0.029 |
| rs7141420 | 0.024 | 0.003 | 0.053 | 0.020 | rs17001654 | 0.031 | 0.005 | 0.054 | 0.036 |
| rs13078960 | 0.030 | 0.004 | -0.0087 | 0.025 | rs11191560 | 0.031 | 0.005 | -0.00024 | 0.020 |
| rs10968576 | 0.025 | 0.003 | 0.030 | 0.021 | rs1528435 | 0.018 | 0.003 | -0.020 | 0.021 |
| rs17024393 | 0.066 | 0.009 | 0.0091 | 0.057 | rs1000940 | 0.019 | 0.003 | -0.0093 | 0.022 |
| rs12429545 | 0.033 | 0.005 | 0.0066 | 0.029 | rs2033529 | 0.019 | 0.003 | 0.028 | 0.020 |
| rs13107325 | 0.048 | 0.007 | 0.024 | 0.040 | rs11583200 | 0.018 | 0.003 | -0.025 | 0.021 |
| rs11165643 | 0.022 | 0.003 | 0.022 | 0.020 | rs9400239 | 0.019 | 0.003 | 0.013 | 0.020 |
| rs17405819 | 0.022 | 0.003 | -0.015 | 0.021 | rs10733682 | 0.017 | 0.003 | -0.0033 | 0.020 |
| rs1016287 | 0.023 | 0.003 | 0.010 | 0.022 | rs11688816 | 0.017 | 0.003 | -0.0025 | 0.033 |
| rs4256980 | 0.021 | 0.003 | 0.014 | 0.021 | rs11057405 | 0.031 | 0.006 | 0.073 | 0.034 |
| rs12401738 | 0.021 | 0.003 | 0.0032 | 0.020 | rs11727676 | 0.036 | 0.006 | 0.14 | 0.020 |
| rs205262 | 0.022 | 0.004 | -0.014 | 0.022 | rs3849570 | 0.019 | 0.003 | 0.028 | 0.023 |
| rs12940622 | 0.018 | 0.003 | 0.027 | 0.020 | rs6477694 | 0.017 | 0.003 | -0.013 | 0.046 |
| rs11847697 | 0.049 | 0.008 | 0.034 | 0.052 | rs7899106 | 0.040 | 0.007 | -0.026 | 0.023 |
| rs2075650 | 0.026 | 0.005 | 0.0062 | 0.029 | rs2176598 | 0.020 | 0.004 | -0.0027 | 0.029 |
| rs2121279 | 0.025 | 0.004 | 0.031 | 0.028 | rs2245368 | 0.032 | 0.006 | -0.013 | 0.023 |
| rs29941 | 0.018 | 0.003 | 0.018 | 0.021 | rs17724992 | 0.019 | 0.004 | -0.0065 | 0.025 |
| rs1808579 | 0.017 | 0.003 | 0.0047 | 0.020 | rs7243357 | 0.022 | 0.004 | -0.042 | 0.023 |
| rs657452 | 0.023 | 0.003 | 0.0088 | 0.020 | rs2033732 | 0.019 | 0.004 | 0.0065 | 0.022 |
| rs12286929 | 0.022 | 0.003 | 0.014 | 0.020 |  |  |  |  |  |

(B) Adult waist-hip ratio (WHR)

|  |  |  |  |  |
| --- | --- | --- | --- | --- |
| RSID | WHR ß | WHR SE | CRC ß | CRC SE |
|  |  |  |  |  |
| rs9491696 | 0.042 | 0.0051 | 0.022 | 0.020 |
| rs6905288 | 0.036 | 0.0044 | 0.039 | 0.022 |
| rs984222 | 0.034 | 0.0041 | 0.0071 | 0.020 |
| rs1055144 | 0.040 | 0.0049 | 0.0054 | 0.024 |
| rs10195252 | 0.033 | 0.004 | 0.019 | 0.020 |
| rs4846567 | 0.034 | 0.0041 | -0.0036 | 0.022 |
| rs1011731 | 0.028 | 0.0034 | -0.0018 | 0.020 |
| rs718314 | 0.030 | 0.0037 | 0.012 | 0.023 |
| rs1294421 | 0.028 | 0.0034 | 0.041 | 0.020 |
| rs1443512 | 0.031 | 0.0038 | -0.042 | 0.023 |
| rs6795735 | 0.025 | 0.0034 | 0.0039 | 0.020 |
| rs4823006 | 0.023 | 0.0034 | -0.012 | 0.020 |
| rs6784615 | 0.043 | 0.0069 | 0.15 | 0.045 |
| rs6861681 | 0.022 | 0.0037 | -0.0063 | 0.021 |

(C) Childhood obesity (CO)

|  |  |  |  |  |
| --- | --- | --- | --- | --- |
| RSID | CO ß | CO SE | CRC ß | CRC SE |
|  |  |  |  |  |
| rs1421085 | 0.36 | 0.043 | 0.018 | 0.020 |
| rs476828 | 0.29 | 0.048 | 0.020 | 0.023 |
| rs12463617 | 0.35 | 0.061 | 0.0087 | 0.026 |
| rs1993709 | 0.32 | 0.051 | 0.031 | 0.026 |
| rs1957894 | 0.41 | 0.084 | 0.000070 | 0.035 |
| rs11208659 | 0.35 | 0.078 | 0.011 | 0.036 |
| rs564343 | 0.20 | 0.038 | -0.048 | 0.020 |
| rs11109072 | 0.51 | 0.111 | 0.076 | 0.052 |
| rs3101336 | 0.19 | 0.042 | -0.0037 | 0.020 |

(D) Birth weight (BW)

|  |  |  |  |  |
| --- | --- | --- | --- | --- |
| RSID | BW ß | BW SE | CRC ß | CRC SE |
|  |  |  |  |  |
| rs900400 | 0.072 | 0.006 | -0.022 | 0.020 |
| rs9883204 | 0.059 | 0.006 | 0.053 | 0.024 |
| rs1042725 | 0.047 | 0.005 | -0.010 | 0.020 |
| rs6931514 | 0.050 | 0.006 | 0.059 | 0.022 |
| rs4432842 | 0.034 | 0.006 | 0.0096 | 0.021 |
| rs724577 | 0.042 | 0.006 | -0.0013 | 0.022 |
| rs1801253 | 0.041 | 0.007 | -0.0069 | 0.023 |

**Supplementary Table 2: Point estimates, confidence intervals, and P-values for regression variables from inverse variance weighted (IVW) and MR-Egger methods.** In IVW, a steeper slope implies a greater degree of pleiotropy. For MR-Egger, the intercept represents the average pleiotropic effect, and an intercept different from zero implies directional pleiotropy.

|  | IVW | | MR-Egger | | |
| --- | --- | --- | --- | --- | --- |
|  | Slope estimate  (95% CI) | P-value |  | Estimate (95% CI) | P-value |
| BMI | 0.14 (-0.15;0.43) | 0.33 | intercept | -0.01 (-0.03;0.01) | 0.40 |
|  |  |  | slope | 0.49 (-0.38;1.36) | 0.26 |
| WHR | 0.36 (-0.14;0.86) | 0.14 | intercept | -0.05 (-0.13;0.03) | 0.22 |
|  |  |  | slope | 1.87 (-0.71;4.44) | 0.14 |
| CO | 0.03 (-0.04;0.1) | 0.38 | intercept | -0.07 (-0.14;0.01) | 0.06 |
|  |  |  | slope | 0.24 (0.00;0.49) | 0.05 |
| BW | 0.17 (-0.4;0.74) | 0.50 | intercept | 0.02 (-0.12;0.16) | 0.73 |
|  |  |  | slope | -0.2 (-2.91;2.51) | 0.85 |

**Supplementary Table 3: MR results of adiposity traits on CRC risk stratified by sex.**

|  |  | |  | |  | |  | |
| --- | --- | --- | --- | --- | --- | --- | --- | --- |
| Sex | BMI | | WHR | | Childhood obesity | | Birth Weight | |
|  | O.R. | *P* value | O.R. | *P* value | O.R. | *P* value | O.R. | *P* value |
|  |  |  |  |  |  |  |  |  |
|  |  |  |  |  |  |  |  |  |
| Male | 1.23  (0.92-1.65) | 0.17 | 2.13  (1.18-3.87) | 0.013 | 1.05  (0.97-1.15) | 0.21 | 1.18  (0.73-1.91) | 0.50 |
| Female | 1.26  (0.92-1.72) | 0.15 | 1.19  (0.63-2.24) | 0.60 | 1.09  (1.00-1.19) | 0.057 | 1.39  (0.83-2.32) | 0.21 |
|  |  |  |  |  |  |  |  |  |
